# CXCR2 knockdown reduces ERK and AKT signaling and migration in PANC-1 pancreatic cancer cells

**DOI:** 10.17912/micropub.biology.002330

**Published:** 2026-09-23

**Authors:** Jacob Krzeminski, Irene K. Guttilla Reed

**Affiliations:** 1 Biology, University of Saint Joseph

## Abstract

Pancreatic cancer mortality is primarily driven by metastatic disease, and epithelial-mesenchymal transition (EMT) is one mechanism that promotes this process. The chemokine receptor CXCR2 has been implicated in tumor progression, but its role in pancreatic cancer remains unclear. We investigated CXCR2 in HPAF-II (epithelial-like) and PANC-1 (mesenchymal-like) pancreatic cancer cells. siRNA-mediated CXCR2 knockdown reduced ERK and AKT phosphorylation and decreased PI3K subunit expression in PANC-1 cells, but not HPAF-II cells. Furthermore, CXCR2 knockdown significantly reduced wound closure in PANC-1 cells. These findings indicate that CXCR2 may regulate EMT-associated signaling and migration in mesenchymal-like pancreatic cancer cells.

**Figure 1. CXCR2 knockdown is associated with decreased PI3K/ERK signaling and reduced wound closure in PANC-1 cells:** **A)** Baseline expression of epithelial (E-cadherin, EPCAM), mesenchymal (vimentin), and EMT-associated (ZEB1, ZEB2) proteins (left) in biological duplicates of PANC-1 and HPAF-II cells. Epithelial markers are expressed at higher levels in HPAF-II cells, and mesenchymal/EMT markers are expressed at higher levels in PANC-1 cells. Proteins associated with activation of the PI3K (top right) and ERK (bottom right) pathways were also expressed at higher levels in PANC-1 cells. **B)** Knockdown of CXCR2 mRNA (left) and protein (right) following transfection with siRNA targeting CXCR2 or a scramble siRNA. **C)** Proteins associated with PI3K and ERK activation decreased following transfection with CXCR2 siRNA. **D)** Wound closure was impaired following transfection with CXCR2 siRNA compared to vehicle (mock) and scramble siRNA treatments. Scale bar = 100 µm. Wound closure was significantly impaired at both the 24 and 48 hour time points **(E)** , * *p* < 0.05 for siRNA treated cells vs. mock or scramble treatments. β-actin was measured as a loading control for protein, and β-tubulin was measured as a loading control for RNA.

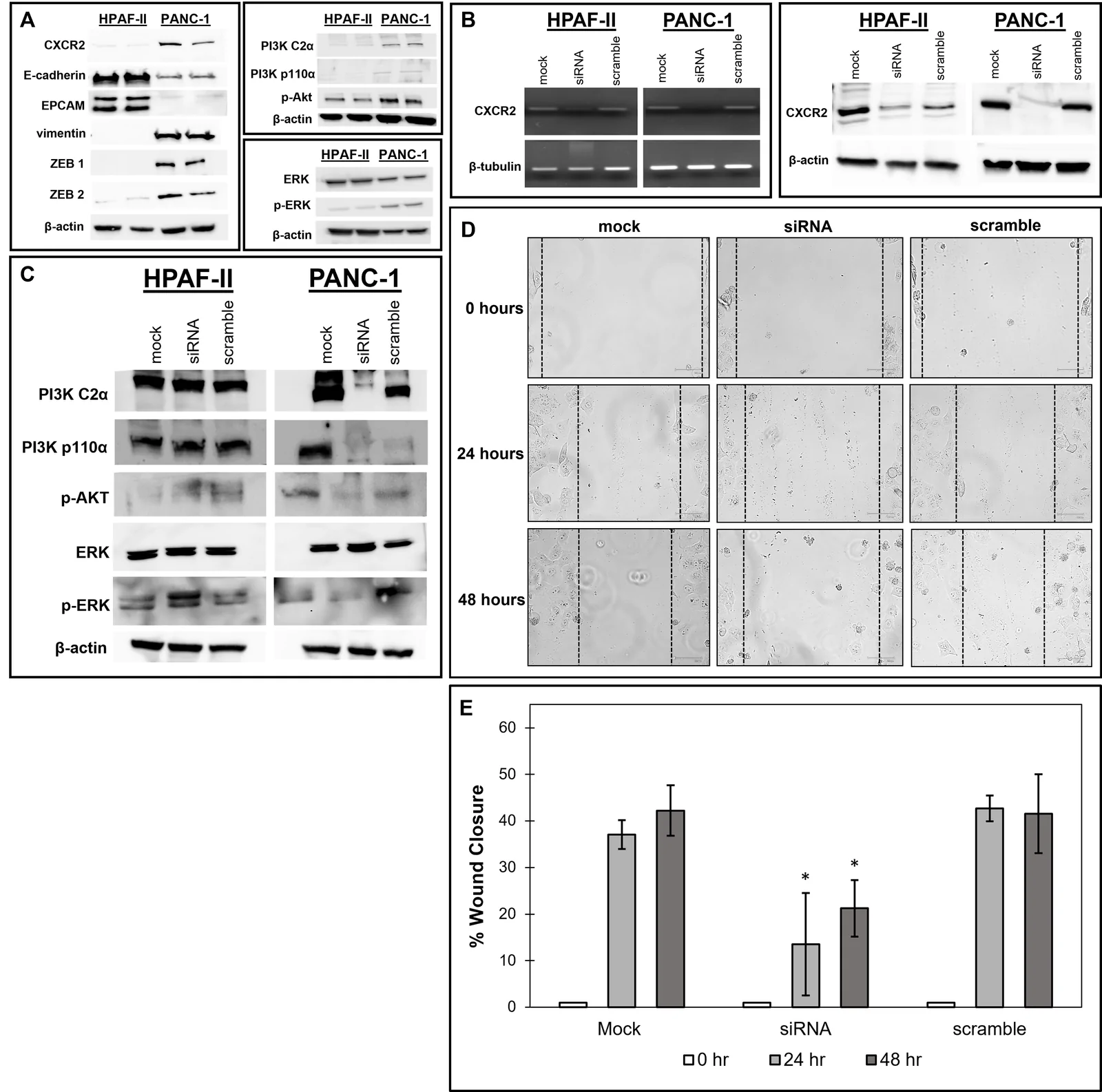



## Description

Pancreatic cancer is one of the deadliest cancers, with survival decreasing significantly once the disease becomes metastatic (Wood et al., 2022). Metastasis is often associated with increased tumor cell migration and invasion, processes that are linked to epithelial-mesenchymal transition (EMT). During EMT, epithelial cells lose features such as E-cadherin expression and gain mesenchymal characteristics, including increased vimentin expression and enhanced motility. Because EMT can promote invasive behavior, identifying upstream signals that regulate this transition may help clarify mechanisms of pancreatic cancer progression.

CXCR2, a chemokine receptor, and one of its ligands CXCL5 are important in recruiting neutrophils to inflammatory sites or injured tissue, but also play a role in cancer progression (Deng et al., 2022). Upregulated levels of CXCL5 and CXCR2 contribute to the progression of pancreatic cancer and have been associated with poor outcomes (Wu et al., 2020; Gautam et al., 2022; Korbecki et al., 2022; Wang et al., 2023). Specifically, elevation in CXCL5 expression has been associated with poor patient prognosis, advanced tumor stages, and decreased overall survival (Li et al., 2011; Wu et al., 2020; Zhang et al., 2020). Pharmacological inhibition or knockdown of CXCR2 reduced angiogenesis, tumor growth, and metastatic progression in pancreatic cancer models, supporting a role for this receptor in disease progression (Donahue and Hines, 2009; Steele et al., 2016; Prajapati et al., 2023). In other cancer types, such as colon and nasopharyngeal cancers, the CXCL5/CXCR2 axis has been linked to activation of downstream signaling pathways, including ERK and PI3K/AKT. The ERK and PI3K/AKT pathways are interconnected signaling cascades that can cooperate to regulate cell survival, proliferation, and motility. Crosstalk between these pathways can promote EMT through regulation of EMT-associated transcription factors and changes in cell adhesion and cytoskeletal dynamics, thereby contributing to tumor cell migration and invasion. Specifically, these pathways can activate EMT-associated transcription factors such as Snail (SNAI1) through regulation of GSK-3β (Zhao et al., 2017; Qiu et al., 2018). This suggests a possible mechanism by which signaling through CXCR2 could promote EMT and migration. Despite these findings, the downstream pathways regulated by CXCR2 and the contribution of CXCR2 signaling to EMT-associated changes in pancreatic cancer have not been fully characterized.

In order to establish a baseline for protein expression of CXCR2 and EMT-associated markers in pancreatic cancer cells, we examined epithelial vs. mesenchymal markers, as well as proteins associated with the ERK and PI3K pathways in HPAF-II and PANC-1 cells. HPAF-II and PANC-1 cells were both derived from patients with pancreatic adenocarcinoma and have metastatic potential; however, HPAF-II cells are well differentiated, whereas PANC-1 cells are poorly differentiated (Deer et al., 2010). Further characterization of these cell lines has demonstrated distinct expression of cellular markers, with HPAF-II cells exhibiting an epithelial phenotype and PANC-1 cells exhibiting a mesenchymal phenotype (Shichi et al., 2022). Consistent with these previously described phenotypes, our analysis demonstrated distinct patterns of EMT-associated marker expression between the two cell lines. HPAF-II cells expressed higher levels of the epithelial markers E-cadherin and EPCAM, whereas PANC-1 cells exhibited increased expression of the mesenchymal marker vimentin and the EMT-associated transcription factors ZEB1 and ZEB2 ( Figure 1A ). E-cadherin and EPCAM are cell-surface proteins that contribute to cell-cell adhesion and are associated with an epithelial phenotype, whereas vimentin is an intermediate filament protein involved in cellular structure and plasticity that is characteristic of a mesenchymal phenotype. Reasoning that ERK and PI3K signaling may be enhanced in the mesenchymal-like cells, we examined the expression of p-AKT, ERK, and p-ERK, as well as the C2α and p110α subunits of PI3K. All of these proteins were detected in both cell lines, with higher expression observed in PANC-1 cells compared with HPAF-II cells ( Figure 1A ).

To determine whether CXCR2 can regulate these pathways, CXCR2 expression was decreased using siRNA. PCR and western blot analysis confirmed decreased CXCR2 mRNA and protein expression compared to mock and scrambled controls in both HPAF-II and PANC-1 cells ( Figure 1B ). Following CXCR2 knockdown, expression of PI3K p110α, p-AKT, and p-ERK was reduced in PANC-1 cells compared with control treatments, while total ERK expression remained relatively the same as baseline ( Figure 1C ). With the exception of an increase in p-ERK, PI3K and ERK pathway proteins were unchanged in HPAF-II cells ( Figure 1C ). These findings suggest that the effects of CXCR2 knockdown on PI3K/AKT and ERK-associated signaling may differ between the mesenchymal-like PANC-1 and epithelial-like HPAF-II cell lines.

To investigate whether the changes in ERK and PI3K pathway proteins observed following CXCR2 knockdown in PANC-1 cells were accompanied by changes in cell migration, a wound healing assay was performed. PANC-1 cells treated with CXCR2 siRNA exhibited delayed wound closure compared to mock and scrambled controls ( Figure 1D ). Quantification of wound closure in PANC-1 cells demonstrated a statistically significant reduction in migration at both 24 and 48 hours following CXCR2 knockdown compared with the mock and scrambled siRNA controls ( Figure 1E ).

The role of the CXCL5/CXCR2 axis in regulating EMT has been more extensively studied in other cancer types. For instance, in colorectal cancer, CXCR2 promoted EMT and cell invasion through activation of both the ERK/Elk-1/Snail and AKT/GSK-3β signaling pathways (Zhao et al., 2017). Similar examples have been reported in nasopharyngeal cancer, where overexpression of CXCL5 and CXCR2 enhanced migration and metastasis through ERK/GSK-3β/Snail signaling, while inhibition of either protein reduced migration and invasion (Qiu et al., 2018). Activation of CXCL5 signaling has also been shown to induce EMT through ERK or PI3K/AKT signaling in bladder, breast, gastric, and hepatocellular carcinomas (Hsu et al., 2013; Gao et al., 2015; Zhou et al., 2015; Mao et al., 2020). In more recent studies, it was demonstrated that CXCL5 overexpression in pancreatic cancer cells increased migration, invasion, and expression of the EMT-associated transcription factors SNAI2 and TWIST, although the signaling pathways involved were not investigated (Wang et al., 2023). 

Ongoing early-phase clinical trials are investigating CXCR2 inhibition as a potential strategy to improve responses to immunotherapy in pancreatic cancer; however, clinical evidence remains limited. Our findings provide additional evidence supporting a role for CXCR2 in pancreatic cancer progression and its potential relevance as a therapeutic target or clinical marker. Knockdown of CXCR2 reduced expression of proteins associated with ERK and PI3K signaling and significantly impaired wound closure in PANC-1 cells, suggesting that CXCR2 contributes to activation of these pathways in pancreatic cancer. Together with previous studies demonstrating that CXCL5 promotes EMT and metastatic behavior, our results support the hypothesis that the CXCL5/CXCR2 axis regulates pancreatic cancer cell migration through ERK- and PI3K/AKT-dependent signaling. While additional experiments are required to directly determine whether these signaling changes regulate EMT, our findings further support a role for CXCR2 in regulating these pathways in pancreatic cancer. 

Several limitations should be considered when interpreting these findings. Data from the wound healing assay were only obtained from the PANC-1 cells due to the implication of ERK and PI3K pathway regulation. Although no changes in ERK or PI3K signaling were observed in HPAF-II cells, making an effect on migration less likely, this possibility should still be evaluated experimentally. In addition, because the wound healing assay was performed in complete growth medium, the contribution of cell proliferation to wound closure cannot be excluded. However, preliminary data showed no significant differences in PANC-1 cell proliferation following CXCR2 siRNA transfection compared with mock and scrambled siRNA controls, suggesting that proliferation was not the primary contributor to the observed reduction in wound closure. The present study was also limited to siRNA-mediated knockdown of CXCR2 in two pancreatic cancer cell lines. Knockdown of CXCL5 independently or in conjunction with CXCR2 was not investigated, preventing direct comparison of ligand and receptor inhibition. Although these findings were observed across more than one independent experiment, additional experimental replicates are needed to confirm the observed effects of CXCR2 knockdown on ERK and PI3K/AKT signaling. In addition, measurement of total AKT would allow changes in AKT activation to be evaluated by comparing phosphorylated and total AKT levels. Future studies should determine whether inhibition of CXCL5 produces similar effects on ERK and PI3K/AKT signaling, and further investigate downstream mediators including GSK-3β, Snail, and other EMT-associated transcription factors. Additional functional assays, including invasion and three-dimensional migration models, would further clarify the role of the CXCL5/CXCR2 axis in pancreatic cancer progression and metastasis.

## Methods

*Cell culture*

HPAF-II cells were cultured in EMEM (ATCC) and PANC-1 cells were cultured in DMEM (ATCC) supplemented with 10% fetal bovine serum (ThermoFisher Scientific) and 1% penicillin/streptomycin (ThermoFisher Scientific). All cells were incubated at 37°C in 5% CO _2_ and passaged as needed. Passage numbers for both cell types ranged from one to twelve in all experiments.

*siRNA knockdown of CXCR2*

CXCR2 was transiently knocked down using 50 nM *Silencer* ^Ⓡ^ CXCR2 siRNA. The CXCR2 siRNA sequences were: Sense - 5' CCGUCUACUCAUCCAAUGUtt 3'; Antisense - 5' ACAUUGGAUGAGUAGACGGtc 3'. The siRNA sequences used for the scramble treatment (Negative Control No. 4 siRNA) are proprietary. Cells were seeded in 6-well plates and grown to 60 - 80% confluency. For transfection of two wells, 20 µL of 10 µM CXCR2 siRNA was diluted in 500 µL Opti-MEM, and 10 µL Lipofectamine RNAiMAX was separately diluted in 500 µL Opti-MEM. The diluted siRNA and Lipofectamine RNAiMAX were combined and incubated for 5 minutes to allow complex formation. Each well contained 1.5 mL Opti-MEM and received 500 µL of the siRNA/Lipofectamine RNAiMAX complex, corresponding to 10 µL siRNA and 5 µL Lipofectamine RNAiMAX per well and a final siRNA concentration of 50 nM. Cells were incubated in transfection medium for 4 hours, after which complete growth medium was restored. RNA was harvested 24 hours post-transfection, and protein was harvested 48 hours post-transfection.

*cDNA synthesis and polymerase chain reaction*

To measure CXCR2 mRNA expression following siRNA knockdown, total RNA was isolated from cells grown in 6-well plates using 1 mL TRIzol Reagent (Invitrogen) per well according to the manufacturer’s instructions and quantified using a NanoDrop OneC spectrophotometer. cDNA was synthesized from 2 µg of total RNA according to the SuperScript IV protocol (ThermoFisher Scientific). PCR was performed in a total reaction volume of 25 µL containing 12.5 µL One *Taq* Hot Start Master Mix (New England Biolabs), 0.5 µL of each 10 µM forward and reverse primer, and 2 µL of cDNA. CXCR2 was amplified using the forward primer 5′-TCACATTCCAAGCCTCATGTCC-3′ and reverse primer 5′-GCAGAGCTCCAGCAAATGACATA-3′. β-tubulin was used as a control and amplified using the forward primer 5′-GCAGAACAAGAACAGCAGCT-3′ and reverse primer 5′-GGTGAACTCCATCTCGTCCA-3′. PCR products were resolved on a 2% agarose gel stained with SYBR Safe and imaged using a Bio-Rad ChemiDoc imaging system.

*Western blot*

Total protein was isolated from cells grown in 6-well plates using 250 µL RIPA buffer containing 1X Halt™ Protease Inhibitor Cocktail (ThermoFisher Scientific). Protein concentrations were determined using a Qubit fluorometer. For each sample, 20 - 40 µg of total protein was combined with 2X Laemmli buffer and resolved on a 10% SDS-PAGE gel, then transferred to 0.2 µm nitrocellulose using the Trans-Blot Turbo System (Bio-Rad) with the Mixed MW protocol. Protein loading amounts were kept consistent within each experiment. Membranes were blocked in 5% milk in TBST for one hour at room temperature, then incubated with primary antibodies overnight at 4°C in 5% milk in TBST, followed by three 5-minute washes in TBST. Membranes were then incubated with HRP-conjugated secondary antibodies for 1 hour at room temperature and washed three additional times for 5 minutes each in TBST. Proteins were detected using Clarity ECL substrate and imaged using a Bio-Rad ChemiDoc imaging system.

*Wound healing assay*

Cells were seeded in 6-well plates and grown to confluence. Then, media was removed, and cells were treated with the siRNA protocol. After incubation with siRNA for 24 hours, a wound was created in each well using a P-10 micropipette tip. Images were taken at 24-hour intervals using the EVoS Floid microscope to measure progress of wound closure. All treatments were repeated in triplicate. Percent wound closure was calculated for three independent trials for each treatment, and data are presented as the mean ± standard deviation. Statistical significance was assessed separately at 24 and 48 hours using one-way ANOVA followed by planned pairwise comparisons of the CXCR2 siRNA-treated group with the mock and scramble controls, with Bonferroni correction for multiple comparisons. Differences were considered statistically significant at p < 0.05.

## Reagents

**Table:** 

**Reagents**	**Catalog #**	**Animal**	**Dilution**	**Clonality**	**Available from**
HPAF-II cells	CRL-1997	human	N/A	N/A	American Type Culture Collection (ATCC)
PANC-1 cells	CRL-1469	human	N/A	N/A	American Type Culture Collection (ATCC)
*Silencer* ^Ⓡ^ CXCR2 siRNA	AM16708	human	50 nM	N/A	ThermoFisher Scientific
Invitrogen *Silencer* ^TM^ Negative Control No. 4 siRNA (scramble)	AM4641	human	50 nM	N/A	ThermoFisher Scientific
CXCR2 antibody (GT547)	20634-1-AP	rabbit	1:500	polyclonal	Proteintech
p-ERK(1/2) antibody	4370T	rabbit	1:500	monoclonal	Cell Signaling Technology
ERK 1/2 antibody	4695T	rabbit	1:500	monoclonal	Cell Signaling Technology
E-cadherin antibody	3195T	rabbit	1:500	monoclonal	Cell Signaling Technology
Vimentin antibody	5741T	rabbit	1:500	monoclonal	Cell Signaling Technology
ZEB1 antibody	70512	rabbit	1:250	monoclonal	Cell Signaling Technology
ZEB2 antibody (E-11)	sc-271984	mouse	1:500	monoclonal	Santa Cruz Biotechnology
PI3K C2α antibody (G-5)	sc-365290	mouse	1:500	monoclonal	Santa Cruz Biotechnology
PI3K p110α antibody (E-7)	sc-518070	mouse	1:250	monoclonal	Santa Cruz Biotechnology
p-Akt 1/2/3 antibody (C-11)	sc-514032	mouse	1:500	monoclonal	Santa Cruz Biotechnology
EPCAM antibody (323/A3)	sc-73491	mouse	1:500	monoclonal	Santa Cruz Biotechnology
β-actin antibody (C-4)	sc-47778	mouse	1:1000	monoclonal	Santa Cruz Biotechnology
anti-rabbit IgG (H+L)-HRP conjugate (secondary antibody)	170-6515	goat	1:2000	polyclonal	Bio-Rad
anti-mouse IgG (H+L)-HRP conjugate (secondary antibody)	170-6516	goat	1:2000	polyclonal	Bio-Rad
